# A Comprehensive Analysis of Baseline Clinical Characteristics and Biomarkers Associated with Outcome in Advanced Melanoma Patients Treated with Pembrolizumab

**DOI:** 10.3390/cancers13020168

**Published:** 2021-01-06

**Authors:** Gil Awada, Yanina Jansen, Julia Katharina Schwarze, Jens Tijtgat, Lennert Hellinckx, Odrade Gondry, Sim Vermeulen, Sarah Warren, Kelly Schats, Pieter-Jan van Dam, Mark Kockx, Marleen Keyaerts, Hendrik Everaert, Teofila Seremet, Anne Rogiers, Bart Neyns

**Affiliations:** 1 Department of Medical Oncology, Universitair Ziekenhuis Brussel, 1090 Brussels, Belgium; gil.awada@uzbrussel.be (G.A.); juliakatharina.schwarze@uzbrussel.be (J.K.S.); jens.tijtgat@uzbrussel.be (J.T.); lennert.hellinckx@vub.be (L.H.); teofila.caplanusi@uzbrussel.be (T.S.); 2 Department of Surgery, Universitair Ziekenhuis Brussel, 1090 Brussels, Belgium; yanina.jansen@uzbrussel.be; 3 Department of Nuclear Medicine, Universitair Ziekenhuis Brussel, 1090 Brussels, Belgium; odrade.gondry@uzbrussel.be (O.G.); sim.vermeulen@uzbrussel.be (S.V.); marleen.keyaerts@uzbrussel.be (M.K.); hendrik.everaert@uzbrussel.be (H.E.); 4 NanoString Technologies, Seattle, WA 98109, USA; swarren@nanostring.com; 5 HistoGeneX, 2610 Antwerp, Belgium; kelly.schats@histogenex.com (K.S.); pieter-jan.vandam@histogenex.com (P.-J.v.D.); mark.kockx@histogenex.com (M.K.); 6 Department of Psychiatry, Centre Hospitalier Universitaire Brugmann, 1020 Brussels, Belgium; anne.rogiers@chu-brugmann.be

**Keywords:** advanced melanoma, biomarkers, pembrolizumab, immunotherapy, multivariate analysis

## Abstract

**Simple Summary:**

Pembrolizumab, a monoclonal antibody targeting programmed cell death 1, improves the survival of patients with advanced melanoma. This study aimed to investigate the association of baseline clinical characteristics, laboratory and imaging variables, and gene expression profiling scores on tumor tissue analysis of advanced melanoma patients who were treated with pembrolizumab, with survival using univariate and multivariate analysis. Baseline organ function (reflected by the presence of active brain metastases, number of metastatically affected organs, albumin) and systemic inflammatory/immunologic status (reflected by albumin, C-reactive protein, absolute lymphocyte count, neutrophil-to-lymphocyte ratio) are the most important clinical and/or laboratory parameters predictive of survival. Novel biomarkers include the baseline presence of *BRAF^V600^* or *NRAS^Q61/G12/G13^* mutant circulating tumor DNA and baseline total metabolic tumor volume assessed by whole-body ^18^F-FDG-PET/CT. Gene expression profiling scores by the NanoString PanCancer IO360 panel were not conclusive in our patient population.

**Abstract:**

Background: Pembrolizumab improves the survival of patients with advanced melanoma. A comprehensive analysis of baseline variables that predict the benefit of pembrolizumab monotherapy has not been conducted. Methods: Survival data of patients with advanced melanoma who were treated with pembrolizumab in a single university hospital were collected. A multivariate Cox regression analysis was performed to correlate baseline clinical, laboratory, and radiologic characteristics and NanoString IO360 gene expression profiling (GEP) with survival. Results: 183 patients were included (stage IV 85.2%, WHO performance status ≥1 31.1%; pembrolizumab first-line 25.7%), of whom 112 underwent baseline ^18^F-FDG-PET/CT imaging, 58 had circulating tumor DNA (ctDNA) assessments, and GEP was available in 27 patients. Active brain metastases, a higher number of metastatic sites, lower albumin and absolute lymphocyte count (ALC), higher C-reactive protein (CRP) and neutrophil-to-lymphocyte ratio, higher total metabolic tumor volume (TMTV), and higher ctDNA levels were associated with worse survival. Elevated lactate dehydrogenase (LDH) ≥ 2ULN (upper limit of normal), CRP ≥ 10ULN, or ALC < 750/mm^3^ delineate a subpopulation where treatment with pembrolizumab is futile. A TMTV ≥ 80 mL encompassed 17/21 patients with LDH ≥ 2ULN, CRP ≥ 10ULN, or ALC < 750/mm^3^. No significant associations were observed between baseline GEP scores and survival. Conclusion: Multiple baseline variables correlate with survival on pembrolizumab. TMTV is a more comprehensive baseline biomarker than CRP, LDH, or ALC in predicting the futility of pembrolizumab.

## 1. Introduction

Pembrolizumab and nivolumab, two therapeutic monoclonal antibodies that block the programmed cell death protein 1 (PD-1, CD279) immune checkpoint receptor, improve survival in patients with advanced melanoma when compared to the cytotoxic T-lymphocyte-associated antigen 4 (CTLA-4, CD152) immune checkpoint inhibitor (ICI) ipilimumab or dacarbazine chemotherapy and have become a preferred treatment option [1,2,3]. In several first-line phase 3 clinical trials, the objective response rate (ORR) for pembrolizumab and nivolumab ranges between 42.0 and 45.0%, median progression-free survival (PFS) between 5.1 and 8.4 months, and median overall survival (OS) between 32.7 and 37.3 months. Advanced melanoma patients treated with PD-1 ICI are at highest risk for disease progression during the first 6 months on therapy. After this period, the progression risk gradually decreases and about 23.0–29.0% of patients will remain free from progression at five years following treatment initiation [1,2,3]. Moreover, patients who electively discontinue therapy are at low risk for subsequent progression of disease [4,5,6]. The risk for progression after elective discontinuation likely correlates with the quality of the response, as patients with a complete response (CR) on computed tomography (CT) or complete metabolic response (CMR) on 18-fluorodeoxyglucose positron emission tomography/computed tomography (^18^F-FDG-PET/CT) have the lowest risk of progression, while patients with partial response (PR), stable disease (SD), or non-CMR as best response are more likely to progress during follow-up [4,5,7].

Baseline parameters that correlate with survival on ICI are an expanding area of research. Bearing in mind the specific association of risk for progression or death per time interval, investigating baseline and on-treatment variables that could help predict outcome can be separated into two distinct objectives. First, the upfront identification of patients who derive no or insufficient benefit could spare them from being exposed to futile therapy and from potentially harmful immune-related adverse events. Second, predicting at baseline or early during therapy who will derive a durable qualitative benefit that allows withholding of further therapy with a low risk for subsequent progression or death is another high-value objective.

A variable that has been consistently found to correlate with the success of PD-1 ICI has been the treatment line, with patients who have previously been exposed to ipilimumab or BRAF-/MEK-inhibitors having a less favorable ORR, PFS, and OS [8]. Nevertheless, even pretreated patients can derive a long-term PFS benefit and safely discontinue therapy [4]. The presence of brain metastases is also associated with a poor prognosis and has a significant impact on outcome with ICI. In this particular population, dual checkpoint inhibition targeting PD-1 plus CTLA-4 may be superior to PD-1 ICI alone, at least in terms of PFS [9,10]. In this population, however, disease burden, as assessed by the diameter and number of lesions, neurological symptoms, and associated corticosteroid need, has a profound impact on outcome [11].

Beyond the line of therapy and the presence of brain metastases, prospective large-scale clinical trials so far have only addressed dichotomized baseline clinical and tumor variables and did not report significant differences in outcome from subgroup analyses [12,13,14]. However, when analyzed in greater detail, a correlation between outcome and baseline lactate dehydrogenase (LDH) was found, with patients having a high baseline LDH (arbitrarily, a cutoff of twice the upper limit of normal [ULN]) deriving very little benefit from PD-1 ICI [15,16,17]. Likewise, C-reactive protein (CRP) and the serum levels of interleukin-6 correlate with outcome [16,18]. Using multivariate analysis, an independent association of low blood relative lymphocyte and eosinophil counts, low LDH, and the absence of visceral metastases other than lung and soft tissue metastases with OS has already been reported in melanoma patients who were treated with pembrolizumab monotherapy [19].

Higher baseline tumor burden, reflected by the number of metastatic tumor localizations, baseline tumor size assessed by CT, or total metabolic tumor volume assessed by ^18^F-FDG-PET/CT, has been associated with worse outcome on ICI in multiple studies [20,21,22,23]. In addition, several groups have reported that the presence of baseline and on-treatment circulating tumor DNA (ctDNA) negatively correlates with response and survival [24,25].

Finally, tumor tissue analysis has been given attention as a potential tool for predicting efficacy. Unlike other tumor types, tumor tissue biomarkers, except for *BRAF^V600^* mutational testing, so far have not been useful for implementation in clinical decision-making in advanced melanoma patients. Immunohistochemical (IHC) scoring systems including the 22C3 PD-L1 (programmed cell death ligand 1, CD274) MEL score correlated with outcome in a pooled analysis of patients treated with pembrolizumab [26]. Expression of PD-L1 is upregulated in response to interferon-gamma (IFN-γ) signaling and responsiveness to PD-1 ICI is associated with preexisting IFN-γ-mediated immune activation that includes tumor-specific major histocompatibility complex (MHC) class II expression [27]. Likewise, a T-cell-inflamed gene expression profile (GEP), indicative of a T-cell-activated tumor microenvironment, was associated with the clinical benefit of pembrolizumab [28]. 

Multivariate models incorporating baseline clinical characteristics and variables from laboratory, imaging, and tumor tissue analysis have not been reported so far. In this study, using a prospectively identified large real-world cohort of advanced melanoma patients treated with pembrolizumab, we have built a multivariate model that incorporates clinical, laboratory, radiologic as well as tumor tissue variables (gene expression profiling) in order to identify baseline characteristics that help to predict durable PFS and OS on or are associated with futility of treatment with pembrolizumab monotherapy in patients with advanced melanoma.

## 2. Methods

### 2.1. Study Design, Patients, and Treatment

This single-center explorative analysis involves patients with advanced (unresectable or metastatic, according to the American Joint Committee on Cancer (AJCC) TNM 8th edition) melanoma who were treated with pembrolizumab monotherapy (2 mg/kg every 3 weeks) in the first- or later-line setting in the Universitair Ziekenhuis Brussel (Brussels, Belgium). Only patients with cutaneous or mucosal melanoma or melanoma with an unknown primary lesion were included; patients with uveal melanoma were excluded. Patients who received concomitant palliative radiation therapy and/or underwent surgery during pembrolizumab treatment could also be included in this analysis. All patients provided written informed consent. 

### 2.2. Assessments

All patients underwent a blood test at baseline and during follow-up visits, analyzing at least the differential blood cell count with determination of the absolute lymphocyte (ALC) and neutrophil count (ANC), serum albumin (ALB), CRP, LDH, and liver and renal function tests. Plasma samples for ctDNA were collected in a subset of patients with a known *BRAF^V600^* or *NRAS^Q61/G12/G13^* mutation. Imaging was performed by CT, whole-body ^18^F-FDG-PET/CT (vertex to toes), and/or magnetic resonance imaging (MRI) of the brain, depending on the clinical context and availability.

### 2.3. Response Evaluation and Imaging

Tumor responses were evaluated using the immune-related response criteria (irRC) [29]. PFS was defined as the time between treatment initiation and progressive disease (PD) or death (whichever occurred first); OS was defined as the time between treatment initiation and death. In patients who had undergone baseline whole-body ^18^F-FDG-PET/CT, the total metabolic tumor volume (TMTV) was calculated as the sum of all tumor-associated voxels with a standardized uptake value (SUV) above the mean SUV measured in a reference region in normal liver tissue plus 3 standard deviations of tumor lesions sized ≥ 1 mL (Syngo.via software, Siemens Healthineers GmbH, Erlangen, Germany) [23].

### 2.4. Plasma Mutant Circulating Tumor DNA Analysis

The method of analysis of baseline plasma *BRAF^V600^* and *NRAS^Q61/G12/G13^* mutant ctDNA has been described in a previous article by our group [25]. The baseline evaluation of ctDNA was dichotomized as detectable or undetectable and quantified as copies of mutant ctDNA.

### 2.5. Gene Expression Profiling and PD-L1 Immunohistochemistry

The NanoString PanCancer IO360 panel was used for GEP on RNA (Table S1). Only tumor samples that had been collected before pembrolizumab initiation were taken into consideration for this analysis. Macrodissection was performed for all tumor samples (archival tissue) with the aim to enrich the tumor material and omitting the interference of the normal adjacent tissue in the final read-out as much as possible. Guided by the hematoxylin and eosin stain, on which the pathologist has annotated the tumor area, the adjacent non-tumor tissue is removed by scraping manually using a scalpel. The total RNA of the deparaffinized, macrodissected slides is extracted using the commercial High Pure FFPET RNA isolation kit (Cat N° 03 270 289 001, Roche, Anderlecht, Belgium) in accordance with the kit insert. The total RNA input was 500 ng. Data analysis was performed by NanoString (Seattle, WA, USA). 

Immunohistochemistry (IHC) for PD-L1 was performed to evaluate concurrence with the PD-L1 GEP score. Melanoma samples were immunohistochemically stained using the FDA-approved PD-L1 IHC 22C3 pharmDx assay (Agilent, Santa Clara, CA, USA). All PD-L1 IHC 22C3 pharmDx stainings were performed in a central Clinical Laboratory Improvement Amendments-approved IHC laboratory (HistoGeneX, Antwerp, Belgium) on 3 µm-thick histological sections, as detailed in the product kit insert on Dako Autostainer Link48 autostainers. All immunostained slides and matching hematoxylin and eosin-stained sections were scanned with a Pannoramic 250 Flash III digital scanner (3DHISTECH, Budapest, Hungary) at 20x magnification. Scanned images were uploaded for evaluation into a proprietary web-based digital pathology environment at HistoGeneX with the use of the Pathomation Digital Pathology System (HistoGeneX, Antwerp, Belgium). Digitized slides of the 22C3 PD-L1 assay were scored by a certified pathologist at HistoGeneX (Antwerp, Belgium). Tumor cell immunoreactivity was captured in terms of the tumor proportion score (TPS), which represents the best estimated percentage (0–100%) of viable tumor cells showing partial or circumferential membrane PD-L1 staining at any intensity. 

### 2.6. Statistical Analysis

Descriptive statistics were used to characterize the patient population. Baseline parameters that were taken into account for this research are summarized in Table 1 and were investigated as categorical or both continuous and categorical variables. 

The Kaplan–Meier method was used to determine median PFS and OS (in weeks). The log-rank test was used to compare survival between subgroups. A multivariate Cox proportional hazards regression model was used to investigate the association between baseline parameters and outcome (PFS and OS). Only factors that were significant in the univariate analysis were included in the multivariate analysis. The level of significance was 0.05 (two-sided) in all analyses. A supervised recursive partitioning analysis was performed by exclusion of the most significant parameter in multivariate analysis at each step. IBM SPSS Statistics version 26.0 (Armonk, NY, USA) was used for statistical analysis. The database was locked on 29 March 2020. 

## 3. Results

### 3.1. Baseline Characteristics

A total of 183 consecutive patients with advanced cutaneous melanoma (85.8%), mucosal melanoma (2.7%), or melanoma with an unknown primary lesion (11.5%) who received at least one administration of pembrolizumab (treatment initiated between 1 September 2014 and 3 September 2019) were identified and included in this analysis (“total study population”). Baseline imaging with whole-body ^18^F-FDG-PET/CT was performed in 112 patients (61.2%); data on baseline ctDNA samples were available in 58 patients (31.7%); representative tumor samples (i.e., before pembrolizumab initiation) were available in 27 patients and analyzed for GEP (14.8%). The baseline characteristics of the total study population and of each subgroup are shown in Table 2. 

### 3.2. Treatment Disposition and Efficacy in the Total Study Population

As of 29 March 2020, 89 of 183 patients (48.6%) have died. The median duration of follow-up in the surviving 94 patients is 210.9 weeks (range 29.7–290.3). Three patients (1.6%) are continuing on treatment, 37 patients (20.2%) were or are being treated with a subsequent treatment line, and 54 patients (29.5%) have discontinued pembrolizumab in the absence of PD. Thirteen patients (7.1%) permanently discontinued pembrolizumab for reasons of toxicity, of whom four also had a confirmed response. The median duration of pembrolizumab treatment was 23.4 weeks (range 1.3–199.9). During pembrolizumab treatment, 44 patients (24.0%) received concurrent palliative radiation therapy, and 11 patients (6.0%) underwent surgery with the aim of reducing tumor mass.

Seventy-two patients (39.3%) achieved an objective response (CR: 47 [25.7%]; PR: 25 [13.7%]). Median time to response was 21.1 weeks (range 5.0–142.3). The disease control rate (DCR) was 53.0%. Median PFS was 20.4 weeks (95% confidence interval [95% CI] 10.6–30.3); median OS was 168.4 weeks (95% CI NR-NR) (Figures S1 and S2). 

### 3.3. Baseline Parameters Associated with PFS and OS in the Total Study Population

World Health Organization Performance Status (WHO PS) ≥ 1, tumor stage IV, the presence of active brain metastases (symptomatic or requiring corticosteroids for symptom control), ≥2 metastatic sites, ≥1 prior therapies, ALB < LLN (lower limit of normal), LDH ≥ ULN, CRP ≥ 2ULN, ALC < 750/mm^3^, ANC ≥ 7500/mm^3^, and a neutrophil-to-lymphocyte ratio (NLR) ≥ 5 were associated with worse PFS in univariate analysis (*p* ≤ 0.042) (Table 3). In multivariate analysis, the presence of active brain metastases (hazard ratio [HR] 2.189 [95 CI 1.296–3.696]; *p* = 0.003), ≥2 metastatic sites (HR 1.996 [95% CI 1.296–3.074]; *p* = 0.002), CRP ≥2ULN (HR 2.328; [95% CI 1.601–3.385]; *p* < 0.001), and ALC <750/mm^3^ (HR 2.767 [95% CI 1.485–5.156]; *p* = 0.001]) were associated with worse PFS.

In univariate analysis, WHO PS ≥1, tumor stage IV, the presence of active brain metastases, ≥2 metastatic sites, ≥1 prior therapies, corticosteroid use (≥8 mg of methylprednisolone, or equivalent), ALB < LLN, CRP ≥ 2ULN, LDH ≥ ULN, ALC < 750/mm^3^, ANC ≥ 7500/mm^3^, and an NLR ≥5 were associated with worse OS (*p* ≤ 0.013) (Table 3). In multivariate analysis, the presence of active brain metastases (HR 2.657 [95% CI 1.493–4.729]; *p* = 0.001), ≥2 metastatic sites (HR 2.365 [95% CI 1.340–4.174]; *p* = 0.003), ALB < LLN (HR 2.446 [95% CI 1.298–4.609]; *p* = 0.006), CRP ≥ 2ULN (HR 2.540 [95% CI 1.585–4.069; *p* < 0.001), ALC < 750/mm^3^ (HR 2.822 [95% CI 1.424–5.594]; *p* = 0.003), and NLR ≥ 5 (HR 1.864 [95% CI 1.142–3.044]; *p* = 0.013) were retained as independent covariables that were associated with worse OS.

**Figure 1:**
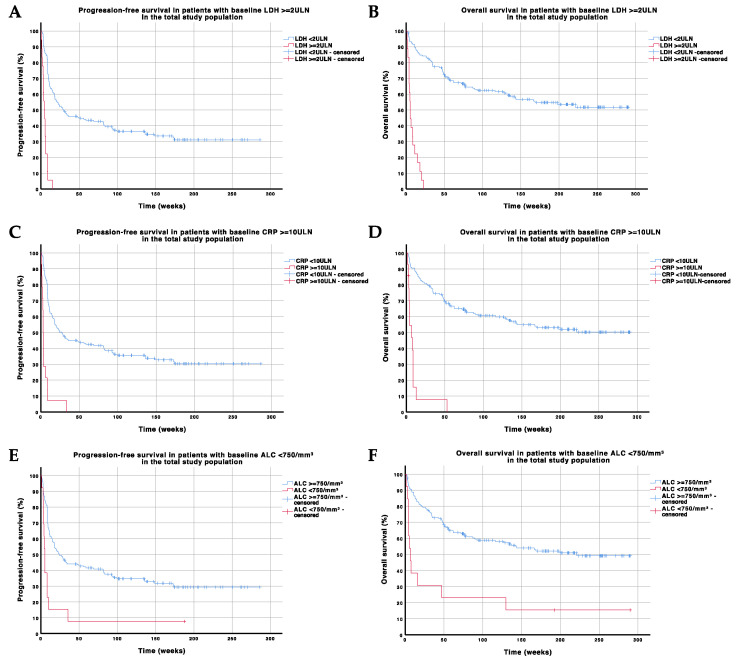
Progression-free and overall survival in subgroups of patients with LDH ≥ 2ULN (panels **A**,**B**), CRP ≥ 10ULN (panels **C**,**D**), and ALC < 750/mm^3^ (panels **E**,**F**) in the total study population. Abbreviations: ALC—absolute lymphocyte count; CRP—C-reactive protein; LDH—lactate dehydrogenase; ULN—upper limit of normal.

**Figure 2:**
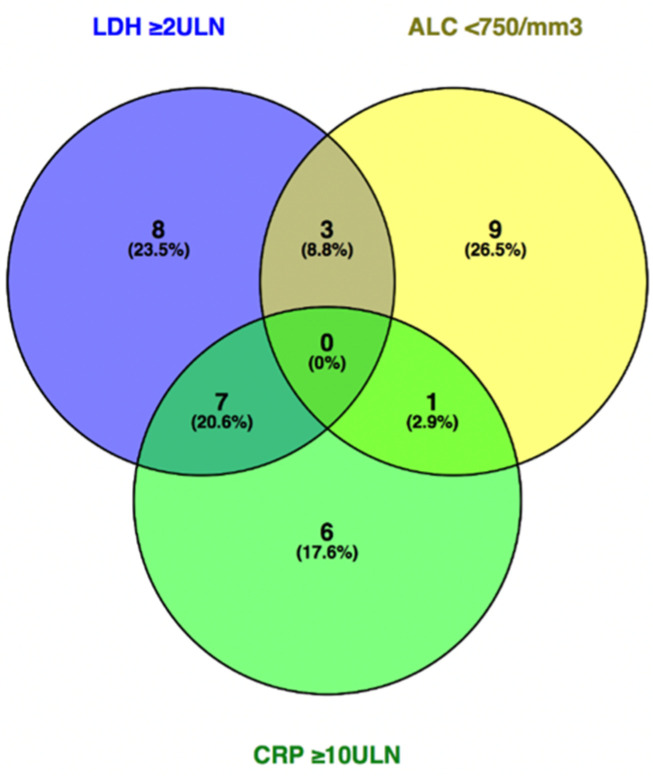
Venn diagram depicting overlap in the number of patients between the categories of LDH ≥ 2ULN (*n* = 18), CRP ≥ 10ULN (*n* = 14) and ALC < 750/mm^3^ (*n* = 13). Abbreviations: ALC—absolute lymphocyte count; CRP—C-reactive protein; LDH—lactate dehydrogenase; ULN—upper limit of normal.

The baseline presence of CRP ≥ 10ULN (*n* = 14), LDH ≥ 2ULN (*n* = 18), or ALC < 750/mm^3^ (*n* = 13) delineates a subpopulation of patients where outcome on pembrolizumab treatment is unfavorable and futile (median PFS < 6 weeks and median OS < 7 weeks) (Figure 1 and Figure 2). A recursive partitioning analysis in the population of patients with CRP < 10ULN, LDH < 2ULN, or ALC ≥ 750/mm^3^ (*n* = 149) with regard to PFS and OS is shown in Figures S3 and S4.

### 3.4. Baseline Parameters Associated with PFS and OS in Patients Who Underwent Baseline Imaging with Whole-Body ^18^F-FDG-PET/CT

The median PFS and OS in the subgroup of 112 patients who underwent baseline imaging with whole-body ^18^F-FDG-PET/CT were 31.0 (95% CI 9.6–52.4) and 221.0 weeks (95% CI NR-NR), respectively (Figures S5 and S6). The ORR was 47.3% and the DCR was 57.1%.

**Figure 3:**
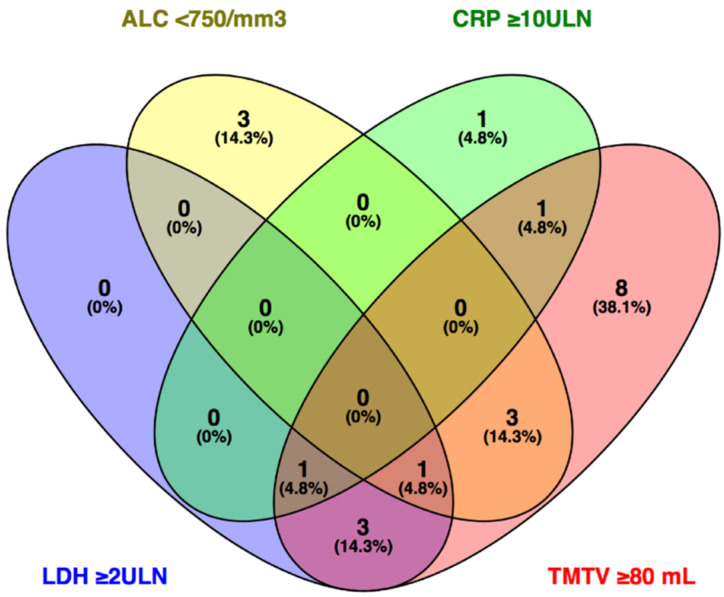
Venn diagram depicting overlap in number of patients between the categories of LDH ≥ 2ULN (*n* = 5), CRP ≥ 10ULN (*n* = 3), ALC < 750/mm^3^ (*n* = 7), and TMTV ≥ 80 mL (*n* = 17). Abbreviations: ALC—absolute lymphocyte count; CRP—C-reactive protein; LDH—lactate dehydrogenase; TMTV—total metabolic tumor volume; ULN—upper limit of normal.

WHO PS ≥ 1, tumor stage IV, the presence of active brain metastases, ≥2 metastatic sites, baseline corticosteroid use, ALB < LLN, LDH ≥ ULN, CRP ≥ 2ULN, ALC < 750/mm^3^, NLR ≥ 5, and TMTV ≥ 80 mL were significantly associated with worse PFS and OS in univariate analysis (*p* ≤ 0.047) (Table 4). Fifteen of seventeen patients (88.2%) with baseline TMTV ≥ 80 mL progressed within 35 weeks and died within 55 weeks; the remaining two patients (11.8%) are free from progression (Figure S7). In this subgroup, TMTV ≥ 80 mL encompassed all five patients with LDH ≥ 2ULN, four of seven patients with ALC < 750/mm^3^, and two of three patients with CRP ≥ 10ULN (Figure 3).

In multivariate analysis, the presence of active brain metastases, ≥2 metastatic sites, ALB < LLN, and the presence of a *BRAF^V600^* mutation were significantly associated with worse PFS (*p* ≤ 0.020); the presence of active brain metastases, ≥2 metastatic sites, ALB < LLN, and ALC < 750/mm^3^ were significantly associated with worse OS (*p* ≤ 0.015). TMTV was associated with worse PFS and OS in multivariate analysis when it was analyzed as a continuous variable (*p* < 0.001). 

### 3.5. Baseline Parameters Associated with PFS and OS in Patients Who Underwent Baseline ctDNA Analysis

The median PFS and OS in the subgroup of 58 patients who underwent baseline analysis of *BRAF^V600^* or *NRAS^Q61/G12/G13^* mutant ctDNA was 9.0 (95% CI 7.4–10.6) and 48.7 weeks (95% CI 0.0–173.1), respectively (Figures S8 and S9). The ORR was 34.5%; the DCR was 43.1%.

In univariate analysis, WHO PS ≥ 1, tumor stage IV, the presence of active brain metastases, ≥2 metastatic sites, ≥1 prior therapies, ALB < LLN, LDH ≥ ULN, CRP ≥ 2ULN, ALC < 750/mm^3^, and NLR ≥ 5 were associated with worse PFS (*p* ≤ 0.039). WHO PS ≥ 1, the presence of active brain metastases, ≥2 metastatic sites, ≥1 prior therapies, ALB < LLN, LDH ≥ ULN, CRP ≥ 2ULN, ALC < 750/mm^3^, ANC ≥ 7500/mm^3^, NLR ≥ 5, and detection of ctDNA were associated with worse OS (*p* ≤ 0.024) (Table 5). In multivariate analysis, the presence of active brain metastases, ≥2 metastatic sites, and ALB < LLN were associated with worse PFS (*p* ≤ 0.039); the presence of active brain metastases, ≥2 metastatic sites, and ALB < LLN were associated with worse OS (*p* ≤ 0.02). ctDNA was only associated with worse OS in multivariate analysis when it was analyzed as a continuous variable (*p* = 0.007).

### 3.6. Baseline Parameters Associated with PFS and OS in Patients Who Underwent Baseline GEP

In the 27 patients who underwent baseline GEP using the NanoString IO360 panel, the median PFS was 51.7 weeks (95% CI 0.0–160.2) (Figure S10); the median OS was not reached (Figure S11). The objective response rate was 40.7%; the disease control rate was 63.0%. PD-L1 IHC was concurrent with PD-L1 GEP score.

In univariate analysis, after dichotomization (below or equal to versus above the median score of the population), a lower PD-L1 GEP score (≤median) was associated with worse PFS (*p* = 0.032); a higher B7-H3 (B7 homolog 3) GEP score (>median) was associated with worse OS (*p* = 0.010) (Table 6). These were not associated with survival in multivariate analysis.

## 4. Discussion

This single-center study investigated the association between baseline clinical and laboratory parameters, TMTV assessed by whole-body ^18^F-FDG-PET/CT, baseline ctDNA, and GEP on tumor tissue and survival (PFS and OS) in a population of 183 patients with advanced melanoma treated with pembrolizumab monotherapy.

The study population consisted of a majority of patients with stage IV-M1c and -M1d disease (more than 60%) who were pretreated with one or more therapies in 74.3% of cases. Efficacy results (PFS, OS, and ORR) were similar to the data obtained in trials with PD-1 ICI in the advanced melanoma setting, even taking into account the different response evaluation criteria (Response Evaluation Criteria in Solid Tumors (RECIST) versus immune-related response criteria) [1,2,3]. The CR rate was higher in our population which could be explained by the application of concurrent radiation therapy and surgery in 24.0% and 6.0% of patients, respectively.

The most important and consistent clinical and laboratory parameters that were associated with survival in our analysis were the presence of active brain metastases, number of affected organs, ALB, CRP, ALC, and NLR. The significance of the presence of active brain metastases, number of affected organs, and ALB reflects the importance of normal organ function for outcome on pembrolizumab. So far, it remains unclear whether LDH and CRP intrinsically reflect a particular tumor biology (respectively, metabolism, the so-called Warburg effect, and the immunosuppressive cytokine secretion profile) and that this biology determines response to ICI or whether they merely reflect the burden of disease, or both [30]. Lactate dehydrogenase, commonly used to predict outcome and incorporated in the current melanoma staging, was only significant in univariate but not in multivariate analysis. However, lower ALB and ALC, and higher CRP and NLR (which reflects the balance between immunosuppressive neutrophils and immune response-promoting lymphocytes) possibly reflect an unfavorable cytokine secretion profile induced by the tumor, leading to a systemic inflammatory state that could mirror relative immune dysfunction and therefore, worse outcome on PD-1 ICI [31]. Baseline LDH ≥ 2ULN, ALC < 750/mm^3^, or CRP ≥ 10ULN is associated with a dismal prognosis on pembrolizumab monotherapy (futility), indicating that alternative treatments such as BRAF-/MEK-inhibitors or combinatorial ICI strategies should be taken into consideration for these patients.

In univariate analysis, a TMTV cutoff at 80 mL defines a subgroup of patients with significantly lower PFS and OS. This cutoff value was not significant in multivariate analysis. However, significance was seen when TMTV was investigated as a continuous variable. With each unit increase in TMTV, the hazard of progression or death increased by a factor of 1.003 and 1.004, respectively. TMTV is possibly a more comprehensive biomarker for assessing metabolically active tumor mass and more informative to predict outcome to pembrolizumab than LDH. In patients with LDH ≥ 2ULN, ALC < 750/mm^3^, CRP ≥ 10ULN, or TMTV ≥ 80 mL, the latter encompassed 17 of 21 patients (81.0%) which suggests a high baseline TMTV as a single biomarker may be more reliable to predict futility. Normal organ function (reflected by the presence/absence of active brain metastases, number of affected organ sites, and ALB) and ALC also remain important prognostic parameters in this investigated subgroup. Future research involving the use of ^18^F-FDG-PET/CT imaging may investigate the effect of reduction in hypermetabolic tumor mass (by using molecular-targeted therapy [BRAF-/MEK-inhibitors], radiation therapy, and/or surgery) prior to the initiation of treatment with ICI therapy on outcome.

Baseline detection of *BRAF^V600^* or *NRAS^Q61/G12/G13^* mutant ctDNA was associated with worse OS. A higher number of baseline mutant ctDNA copies was associated with worse OS in multivariate analysis, which confirms earlier research performed by our and other research groups [24,25].

Our research did not reveal an association between the NanoString IO360 GEP scores and PFS/OS in multivariate analysis, in particular no correlation was found with tumor inflammation, PD-L1, and IFN-γ scores. The association of lower PD-L1 scores with worse PFS in univariate analysis supports previous research which showed that a higher baseline PD-L1 MEL score was associated with better response, PFS, and OS to pembrolizumab [26]. Our study shows that a higher B7-H3 score (which is an inhibitory immune checkpoint) is associated with worse OS in univariate analysis. Blockade of B7-H3 in vitro has been shown to reinvigorate the cytotoxic T-lymphocyte’s activity against melanoma cells; however, there are no clinical applications of ICI targeting B7-H3 yet [32]. The absence of additional GEP scores associated with PFS/OS could be explained by spatial heterogeneity in the tumor biopsy leading to RNA originating from non-cancerous tissue (such as normal lymphoid tissue) being included in the tumor score counts. Furthermore, our research involved a relatively low number of included tumor samples. This merits further research in a larger patient sample and implementation of microdissection of tumor biopsies.

As a future perspective, it would be of great interest to further validate our model in a larger population treated in a first-line setting, and even reassessing the model when patients are in need of a second (or later) line of therapy.

## 5. Conclusions

This study focused on baseline clinical characteristics and biomarkers that predict outcome of patients with advanced melanoma treated with pembrolizumab monotherapy. Baseline organ function (reflected by the presence of active brain metastases, number of affected organs, ALB) and systemic inflammatory/immunologic status (reflected by ALB, CRP, ALC, NLR) are the most important clinical and/or laboratory parameters predictive of survival. High CRP, high LDH, and/or low ALC delineate a population where treatment with PD-1 ICI monotherapy is futile.

Novel biomarkers include the baseline presence of *BRAF^V600^* or *NRAS^Q61/G12/G13^* mutant ctDNA and baseline TMTV assessed by whole-body ^18^F-FDG-PET/CT. The latter may be more informative than LDH, CRP, and ALC to suggest futility to treatment with PD-1 ICI. GEP scores by the NanoString PanCancer IO360 panel were not conclusive in our patient population.

## Figures and Tables

**Table 1:** Baseline parameters investigated in this analysis.

Clinical Factors	Blood Values	Plasma ctDNA	Imaging	Tissue
- Age • - Sex • - World Health Organization Performance Status • - Tumor stage • - Presence of inactive/active brain - metastases • - Number of affected organs • - Number of prior therapies • - Corticosteroid use •	- Albumin • (35–50 g/L) - Lactate dehydrogenase • (313–618 U/L) - C-reactive protein • (<5 mg/L) - Absolute - lymphocyte count • (1200–3500/mm^3^) - Absolute - neutrophil count • (1200–7500/mm^3^) - Neutrophil-to-lymphocyte ratio •	- Detection of *BRAF^V600^* or *NRAS^Q61/G12/G13^* mutant ctDNA •*	- TMTV •*	- *BRAF^V600^* mutation - status • - NanoString IO360 gene expression profiling scores •

Tumor stage was determined by the American Joint Committee on Cancer TNM 8th edition. Active brain metastases are defined as symptomatic brain metastases or brain metastases requiring corticosteroids for symptom control. Corticosteroid use was defined as the use of ≥8 mg of methylprednisolone (or equivalent). Normal institutional laboratory values are shown in the table. • analyzed as a categorical variable; * analyzed as a continuous variable. Abbreviations: ctDNA—circulating tumor DNA; TMTV—total metabolic tumor volume; U/L—units/liter.

**Table 2:** Baseline patient characteristics.

Baseline Patient Characteristics	Total Study Population *n* = 183	Patients with Baseline ^18^F-FDG-PET/CT *n* = 112	Patients with Baseline Mutant ctDNA Analysis *n* = 58	Patients with Baseline Tissue GEP Analysis *n* = 27
Age (median, (range))	60 (24–93)	61 (26–93)	58 (26–82)	63 (36–93)
Sex (*n* (%))				
Male	88 (48.1)	55 (49.1)	28 (48.3)	14 (51.9)
Female	95 (51.9)	57 (50.9)	30 (51.7)	13 (48.1)
Melanoma subtype (*n* (%))				
Cutaneous	157 (85.8)	100 (89.3)	49 (84.5)	24 (88.9)
Mucosal	5 (2.7)	3 (2.8)	1 (1.7)	0 (0)
Unknown primary	21 (11.5)	9 (8.0)	8 (13.8)	3 (11.1)
WHO PS (*n* (%))				
0	126 (68.9)	78 (69.6)	39 (67.2)	20 (74.1)
1	41 (22.4)	23 (20.5)	13 (22.4)	3 (11.1)
2	16 (8.7)	11 (9.8)	6 (10.3)	4 (14.8)
Tumor stage (*n* (%))				
IIIB	6 (3.3)	5 (4.5)	1 (1.7)	0 (0.0)
IIIC	21 (11.5)	14 (12.5)	4 (6.9)	4 (14.8)
IV-M1a	12 (6.6)	9 (8.0)	5 (8.6)	5 (18.5)
IV-M1b	26 (14.2)	19 (17.0)	4 (6.9)	2 (7.4)
IV-M1c	73 (39.9)	45 (40.2)	26 (44.8)	7 (25.9)
IV-M1d	45 (24.6)	20 (17.9)	18 (31.0)	9 (33.3)
Brain metastases (*n* (%))				
Active	21 (11.5)	8 (7.1)	6 (10.3)	5 (18.5)
Inactive	24 (13.1)	12 (10.7)	12 (20.7)	4 (14.8)
Number of affected organs (*n* (%))				
1	63 (34.4)	46 (41.1)	17 (29.3)	11 (40.7)
2–3	68 (37.2)	44 (39.3)	25 (43.1)	10 (37.0)
4–5	37 (20.2)	17 (15.2)	12 (20.7)	4 (14.8)
>5	15 (8.2)	5 (4.5)	4 (6.9)	2 (7.4)
Number of prior therapies (*n* (%))				
0	47 (25.7)	33 (29.5)	7 (12.1)	11 (40.7)
1	65 (35.6)	45 (40.2)	21 (36.2)	8 (29.6)
2	36 (19.7)	18 (16.1)	15 (25.9)	3 (11.1)
3	17 (9.3)	7 (6.3)	8 (13.8)	2 (7.4)
≥4	18 (9.8)	9 (8.0)	7 (12.1)	3 (11.1)
Prior ipilimumab (*n* (%))	89 (48.6)	53 (47.3)	37 (63.8)	10 (37.0)
Prior BRAF-inhibitor monotherapy (*n* (%))	34 (18.6)	17 (15.2)	15 (25.9)	5 (18.5)
Prior BRAF-/MEK-inhibitor (*n* (%))	61 (33.3)	38 (33.9)	32 (55.2)	9 (33.3)
Corticosteroid use (*n* (%))				
Yes	8 (4.5)	3 (2.8)	2 (3.4)	3 (11.1)
No	175 (95.6)	109 (97.3)	56 (96.6)	24 (88.9)
ALB				
<LLN (*n* (%))	17 (9.3)	8 (7.1)	8 (13.8)	3 (11.1)
≥LLN (*n* (%))	166 (90.1)	104 (92.9)	50 (86.2)	24 (88.9)
Median (g/L)	41	41	40	40
LDH				
<ULN (*n* (%))	123 (67.2)	87 (77.7)	37 (63.8)	22 (81.5)
≥ULN (*n* (%))	60 (32.8)	25 (22.3)	21 (36.2)	5 (18.5)
Median (U/L)	513	483	519	491
CRP				
<ULN (*n* (%))	99 (54.1)	66 (58.9)	28 (48.3)	19 (70.4)
≥ULN (*n* (%))	84 (45.9)	46 (41.1)	30 (51.7)	8 (29.6)
Median (mg/L)	4	3	6	3
ALC				
<LLN (*n* (%))	54 (29.5)	29 (25.9)	17 (29.3)	10 (37.0)
Median (/mm^3^)	1629	1703	1706	1386
ANC				
≥ULN (*n* (%))	19 (10.4)	8 (7.1)	3 (5.2)	1 (3.7)
Median (/mm^3^)	4338	4161.5	4374	4298
NLR				
<5 (*n* (%))	147 (80.3)	95 (84.8)	46 (79.3)	19 (70.4)
≥5 (*n* (%))	36 (19.7)	17 (15.2)	12 (20.7)	8 (29.6)
Median	2.81	2.72	2.69	3.01
*BRAF^V600^* status				
Mutant (*n* (%))	96 (52.5)	56 (50.0)	42 (72.4)	16 (59.3)
Wild-type (*n* (%))	87 (47.5)	56 (50.0)	16 * (27.6)	11 (40.7)
TMTV				
<80 (*n* (%))	NA	95 (84.8)	NA	NA
≥80 (*n* (%))	NA	17 (15.2)	NA	NA
Median (mL)	NA	6.77	NA	NA
ctDNA				
Detectable (*n* (%))	NA	NA	27 (46.6)	NA
Undetectable (*n* (%))	NA	NA	31 (53.4)	NA
Median copy number (/mL)	NA	NA	0	NA
PD-L1 IHC				
Median (%)	NA	NA	NA	0.5 •

Tumor stage was determined by the American Joint Committee on Cancer TNM 8th edition. Active brain metastases are defined as symptomatic brain metastases or brain metastases requiring corticosteroids for symptom control. Corticosteroid use was defined as the use of ≥8 mg of methylprednisolone (or equivalent). * These patients were *NRAS^Q61/G12/G13^* mutant. • PD-L1 IHC was available in 11 patients. Abbreviations: ^18^F-FDG-PET/CT—18-fluorodeoxyglucose positron emission tomography/computed tomography; ALB—albumin; ALC—absolute lymphocyte count; ANC—absolute neutrophil count; CRP—C-reactive protein; ctDNA—circulating tumor DNA; LDH—lactate dehydrogenase; LLN—lower limit of normal; NLR—neutrophil-to-lymphocyte ratio; PD-L1 IHC—programmed cell death ligand 1 immunohistochemistry; TMTV—total metabolic tumor volume; U/L—units/liter; ULN—upper limit of normal; WHO PS—World Health Organization Performance Status.

**Table 3:** Association of baseline parameters with progression-free and overall survival using uni- and multivariate analysis in the total study population.

Baseline Parameters	PFS		OS	
	Univariate HR (*p*-Value)	Multivariate HR (95% CI; *p*-Value)	Univariate HR (*p*-Value)	Multivariate HR (95% CI; *p*-Value)
Age (age decade vs. 20–29)	0.006–0.576 (0.448–0.940)	NA	0.049–0.529 (0.467–0.825)	NA
Sex (male vs. female)	0.051 (0.822)	NA	2.747 (0.546)	NA
WHO PS (≥1 vs. 0)	**18.037 (<0.001)**	NS	**35.151 (<0.001)**	NS
Tumor stage (IV vs. III)	**10.494 (0.001)**	NS	**7.946 (0.005)**	NS
Brain metastases				
Inactive vs. absent	0.013 (0.910)	NS	0.270 (0.604)	NS
Active vs. absent	**21.981 (<0.001)**	**2.189 (1.296–3.696; 0.003)**	**33.194 (<0.001)**	**2.657 (1.493–4.729; 0.001)**
Number of affected organs (≥2 vs. 1)	**24.029 (<0.001)**	**1.996 (1.296–3.074; 0.002)**	**22.769 (<0.001)**	**2.365 (1.340–4.174; 0.003)**
Number of prior therapies (≥1 vs. 0)	**8.609 (0.003)**	NS	**6.511 (0.011)**	NS
Corticosteroid use (yes vs. no)	3.289 (0.070)	NA	**6.210 (0.013)**	NS
ALB (<LLN vs. ≥LLN)	**16.815 (<0.001)**	NS	**28.519 (<0.001)**	**2.446 (1.298–4.609; 0.006)**
LDH (≥ULN vs. <ULN)	**24.794 (<0.001)**	NS	**33.761 (<0.001)**	NS
CRP (≥2ULN vs. <2ULN)	**32.777 (<0.001)**	**2.328 (1.601–3.385; <0.001)**	**39.984 (<0.001)**	**2.540 (1.585–4.069; <0.001)**
ALC (<750/mm^3^ vs. ≥750/mm^3^)	**14.995 (<0.001)**	**2.767 (1.485–5.156; 0.001)**	**17.813 (<0.001)**	**2.822 (1.424–5.594; 0.003)**
ANC (≥7500/mm^3^ vs. <7500/mm^3^)	**4.140 (0.042)**	NS	**10.254 (0.001)**	NS
NLR (≥5 vs. <5)	**15.147 (<0.001)**	NS	**32.615 (<0.001)**	**1.864 (1.142–3.044; 0.013)**
*BRAF^V600^* mutation (mutant vs. wild-type)	3.173 (0.075)	NA	0.004 (0.949)	NA

Significant values are marked in bold. Abbreviations: 95% CI—95% confidence interval; ALB—albumin; ALC—absolute lymphocyte count; ANC—absolute neutrophil count; CRP—C-reactive protein; HR—hazard ratio; LDH—lactate dehydrogenase; LLN—lower limit of normal; NA—not applicable; NLR—neutrophil-to-lymphocyte ratio; NS—not significant; vs.—versus; ULN—upper limit of normal; WHO PS—World Health Organization Performance Status.

**Table 4:** Association of baseline parameters with progression-free and overall survival using uni- and multivariate analysis in the population of patients who underwent baseline imaging with whole-body 18F-FDG-PET/CT.

Baseline Parameters	PFS		OS	
	Univariate HR (*p*-Value)	Multivariate HR (95% CI; *p*-Value)	Univariate HR (*p*-Value)	Multivariate HR (95% CI; *p*-Value)
Age (age decade vs. 20–29)	0.000–0.583 (0.445–0.986)	NA	0.462–1.000 (0.326–0.497)	NA
Sex (male vs. female)	0.072 (0.789)	NA	4.000 (0.617)	NA
WHO PS (≥1 vs. 0)	**7.507 (0.006)**	NS	**19.719 (<0.001)**	NS
Tumor stage (IV vs. III)	**7.648 (0.006)**	NS	**3.961 (0.047)**	NS
Brain metastases				
Inactive vs. absent	0.521 (0.470)	NS	0.026 (0.872)	NS
Active vs. absent	**28.894 (<0.001)**	**3.950 (1.749–8.920; 0.001)**	**56.003 (<0.001)**	**8.629 (3.395–21.935; <0.001)**
Number of affected organs (≥2 vs. 1)	**12.101 (0.001)**	**2.165 (1.278–3.668; 0.004)**	**14.867 (<0.001)**	**2.377 (1.184–4.773; 0.015)**
Number of prior therapies (≥1 vs. 0)	3.351 (0.067)	NA	1.245 (0.265)	NA
Corticosteroid use (yes vs. no)	**7.329 (0.007)**	NS	**17.563 (<0.001)**	NS
ALB (<LLN vs. ≥LLN)	**6.371 (0.012)**	**2.581 (1.161–5.736; 0.020)**	**9.452 (0.002)**	**3.444 (1.362–8.708; 0.009)**
LDH (≥ULN vs. <ULN)	**11.541 (0.001)**	NS	**20.528 (<0.001)**	NS
CRP (≥2ULN vs. <2ULN)	**8.974 (0.003)**	NS	**14.961 (<0.001)**	NS
ALC (<750/mm^3^ vs. ≥750/mm^3^)	**4.760 (0.029)**	NS	**12.242 (<0.001)**	**5.036 (2.062–12.299; 0.009)**
ANC (≥7500/mm^3^ vs. <7500/mm^3^)	0.882 (0.348)	NA	0.185 (0.667)	NA
NLR (≥5 vs. <5)	**6.014 (0.014)**	NS	**16.102 (<0.001)**	NS
*BRAF^V600^* mutation (mutant vs. wild-type)	**4.933 (0.026)**	**2.370 (1.441–3.899; 0.001)**	0.017 (0.897)	NA
TMTV				
≥80 vs. <80 mL	**14.466 (<0.001)**	NS	**45.141 (<0.001)**	NS
Absolute value	NA	**1.003 (1.001–1.004; <0.001)**	NA	**1.004 (1.002–1.006; <0.001)**

TMTV was analyzed both as a categorical and as a continuous variable. Significant values are marked in bold. Abbreviations: 95% CI—95% confidence interval; ALB—albumin; ALC—absolute lymphocyte count; ANC—absolute neutrophil count; CRP—C-reactive protein; HR—hazard ratio; LDH—lactate dehydrogenase; LLN—lower limit of normal; NA—not applicable; NLR—neutrophil-to-lymphocyte ratio; NS—not significant; vs.—versus; TMTV—total metabolic tumor volume; ULN—upper limit of normal; WHO PS—World Health Organization Performance Status.

**Table 5:** Association of baseline parameters with progression-free and overall survival using uni- and multivariate analysis in the population of patients who underwent baseline ctDNA analysis.

Baseline Parameters	PFS		OS	
	Univariate HR (*p*-Value)	Multivariate HR (95% CI; *p*-Value)	Univariate HR (*p*-Value)	Multivariate HR (95% CI; *p*-Value)
Age (age decade vs. 20–29)	0.050–1.000 (0.317–0.822)	NA	0.694–1.184 (0.277–0.405)	NA
Sex (male vs. female)	7.092 (0.707)	NA	0.015 (0.902)	NA
WHO PS (≥1 vs. 0)	**5.074 (0.024)**	NS	**6.197 (0.013)**	NS
Tumor stage (IV vs. III)	**6.657 (0.010)**	NS	2.378 (0.123)	NA
Brain metastases				
Inactive vs. absent	2.920 (0.088)	NS	0.048 (0.827)	NS
Active vs. absent	**13.985 (<0.001)**	**2.839 (1.053–7.654; 0.039)**	**12.810 (<0.001)**	**4.935 (1.707–14.264; 0.003)**
Number of affected organs (≥2 vs. 1)	**9.011 (0.003)**	**2.609 (1.21–5.484; 0.011)**	**9.479 (0.002)**	**3.382 (1.198–9.545; 0.021)**
Number of prior therapies (≥1 vs. 0)	**5.012 (0.025)**	NS	**5.028 (0.025)**	NS
Corticosteroid use (yes vs. no)	3.661 (0.056)	NA	3.670 (0.055)	NA
ALB (<LLN vs. ≥LLN)	**10.396 (0.001)**	**3.968 (1.637–9.616; 0.002)**	**13.424 (<0.001)**	**4.227 (1.584–11.285; 0.004)**
LDH (≥ULN vs. <ULN)	**8.815 (0.003)**	NS	**11.452 (0.001)**	NS
CRP (≥2ULN vs. <2ULN)	**7.896 (0.005)**	NS	**7.046 (0.008)**	NS
ALC (<750/mm^3^ vs. ≥750/mm^3^)	**5.236 (0.022)**	NS	**5.061 (0.024)**	NS
ANC (≥7500/mm^3^ vs. <7500/mm^3^)	2.229 (0.135)	NA	**8.293 (0.004)**	NS
NLR (≥5 vs. <5)	**4.261 (0.039)**	NS	**7.857 (0.005)**	NS
*BRAF^V600^* mutation (mutant vs. wild-type)	2.452 (0.117)	NA	5.291 (0.664)	NA
ctDNA				
Detectable vs. undetectable	3.607 (0.058)	NA	**7.482 (0.006)**	NS
Absolute value	NA	NS	NA	**1.000 (1.000–1.000; 0.007)**

ctDNA was analyzed both as a categorical and as a continuous variable. Significant values are marked in bold. Abbreviations: 95% CI—95% confidence interval; ALB—albumin; ALC—absolute lymphocyte count; ANC—absolute neutrophil count; CRP—C-reactive protein; ctDNA—circulating tumor DNA; HR—hazard ratio; LDH—lactate dehydrogenase; LLN—lower limit of normal; NA—not applicable; NLR—neutrophil-to-lymphocyte ratio; NS—not significant; vs.—versus; ULN—upper limit of normal; WHO PS—World Health Organization Performance Status.

**Table 6:** Association of baseline parameters with progression-free and overall survival using uni- and multivariate analysis in the population of patients who underwent baseline gene expression profiling on tumor tissue.

Baseline Parameters	PFS		OS	
	Univariate HR (*p*-Value)	Multivariate HR (95% CI; *p*-Value)	Univariate HR (*p*-Value)	Multivariate HR (95% CI; *p*-Value)
Age (age decade vs. 30–39)	0.010–2.182 (0.140–0.919)	NA	0.167–1.077 (0.299–0.683)	NA
Sex (male vs. female)	0.482 (0.487)	NA	2.717 (0.544)	NA
WHO PS (≥1 vs. 0)	1.179 (0.357)	NA	**5.639 (0.018)**	NS
Tumor stage (IV vs. III)	1.517 (0.417)	NA	2.236 (0.135)	NA
Brain metastases				
Inactive vs. absent	2.140 (0.143)	NA	**6.211 (0.013)**	NS
Active vs. absent	2.010 (0.156)	NA	**5.018 (0.025)**	NS
Number of affected organs (≥2 vs. 1)	3.815 (0.051)	NA	**6.330 (0.012)**	**25.067 (1.480–424.449; 0.026)**
Number of prior therapies (≥1 vs. 0)	1.046 (0.306)	NA	1.425 (0.402)	NA
Corticosteroid use (yes vs. no)	0.019 (0.889)	NA	0.010 (0.921)	NA
ALB (<LLN vs. ≥LLN)	3.606 (0.058)	NA	**3.862 (0.049)**	**36.404 (2.745–482.728; 0.006)**
LDH (≥ULN vs. <ULN)	10.204 (0.754)	NA	2.960 (0.085)	NA
CRP (≥2ULN vs. <2ULN)	**5.588 (0.018)**	NS	2.022 (0.155)	NA
ALC (<750/mm^3^ vs. ≥750/mm^3^)	**6.959 (0.008)**	**7.715 (1.670–35.633; 0.009)**	**9.445 (0.002)**	**6.732 (1.480–424.449; 0.026)**
ANC (≥7500/mm^3^ vs. <7500/mm^3^)	0.130 (0.719)	NA	0.415 (0.520)	NA
NLR (≥5 vs. <5)	**5.977 (0.014)**	NS	**5.116 (0.024)**	NS
*BRAF^V600^* mutation (mutant vs. wild-type)	0.105 (0.746)	NA	0.395 (0.112)	NA
PD-L1 GEP score (≤median vs. >median)	**4.584 (0.032)**	NS	NA	NA
B7-H3 GEP score (>median vs. ≤median)	NA	NA	**6.695 (0.010)**	NS

The GEP scores were analyzed as categorical variables (below or equal to versus above the median score of the population). Significant values are marked in bold. Only GEP scores that were significant in univariate analysis are mentioned in the table. Abbreviations: 95% CI—95% confidence interval; ALB—albumin; ALC—absolute lymphocyte count; ANC—absolute neutrophil count; B7-H3—B7 homolog 3; CRP—C-reactive protein; GEP—gene expression profiling; HR—hazard ratio; LDH—lactate dehydrogenase; LLN—lower limit of normal; NA—not applicable; NLR—neutrophil-to-lymphocyte ratio; NS—not significant; PD-L1; programmed cell death ligand 1; vs.—versus; ULN—upper limit of normal; WHO PS—World Health Organization Performance Status.

## Data Availability

The data presented in this study are available on reasonable request from the corresponding author. The data are not publicly available due to ethical/privacy reasons.

## Supplementary Materials

The following are available online at https://www.mdpi.com/2072-6694/13/2/168/s1, Table S1: Scores in the NanoString PanCancer IO360 gene expression profiling panel, Figure S1: Progression-free survival in the total study population (*n* = 183), Figure S2: Overall survival in the total study population (*n* = 183), Figure S3: Recursive partitioning analysis in the population of patients with CRP < 10ULN, LDH < 2ULN or ALC ≥ 750/mm^3^ (*n* = 149) with regards to progression-free survival. Abbreviations: 95% CI: 95% confidence interval; ALC: absolute lymphocyte count; CRP: C-reactive protein; LDH: lactate dehydrogenase; mPFS: median progression-free survival; PEMBRO 1L: pembrolizumab as first-line treatment; PEMBRO ≥ 2L: pembrolizumab as second-or-later line treatment; ULN: upper limit of normal; w: weeks, Figure S4: Recursive partitioning analysis in the population of patients with CRP < 10ULN, LDH < 2ULN or ALC ≥ 750/mm^3^ (*n* = 149) with regards to overall survival. Abbreviations: 95% CI: 95% confidence interval; ALC: absolute lymphocyte count; CRP: C-reactive protein; LDH: lactate dehydrogenase; mOS: median overall survival; PEMBRO 1L: pembrolizumab as first-line treatment; PEMBRO ≥ 2L: pembrolizumab as second line or later treatment; ULN: upper limit of normal; w: weeks; WHO PS: World Health Organization Performance Status, Figure S5: Progression-free survival in the subgroup of patients who underwent baseline imaging with whole-body ^18^F-FDG-PET/CT (*n* = 112). Abbreviations: ^18^F-FDG-PET/CT: 18-fluorodeoxyglucose positron emission tomography/computed tomography, Figure S6: Overall survival in the subgroup of patients who underwent baseline imaging with whole-body ^18^F-FDG-PET/CT (*n* = 112). Abbreviations: ^18^F-FDG-PET/CT: 18-fluorodeoxyglucose positron emission tomography/computed tomography, Figure S7: Progression-free and overall survival in the subgroup of patients who underwent baseline imaging with whole-body ^18^F-FDG-PET/CT with baseline TMTV of <80 mL (*n* = 95) versus ≥ 80 mL (*n* = 17). Abbreviations: ^18^F-FDG-PET/CT: 18-fluorodeoxyglucose positron emission tomography/computed tomography; TMTV: total metabolic tumor volume, Figure S8: Progression-free survival curve in the subgroup of patients who underwent baseline ctDNA analysis (*n* = 58). Abbreviations: ctDNA: circulating tumor DNA, Figure S9: Overall survival curve in the subgroup of patients who underwent baseline ctDNA analysis (*n* = 58). Abbreviations: ctDNA: circulating tumor DNA, Figure S10: Progression-free survival curve in the subgroup of patients who underwent baseline gene expression profiling on tumor tissue (*n* = 27), Figure S11: Overall survival curve in the subgroup of patients who underwent baseline gene expression profiling on tumor tissue (*n* = 27).
