# Supplementary material for: A Comprehensive Analysis of Baseline Clinical Characteristics and Biomarkers Associated with Outcome in Advanced Melanoma Patients Treated with Pembrolizumab
Source: Cancers (Basel). 2021 Jan 6;13(2):168. doi: 10.3390/cancers13020168 (PMC7825041; doi:10.3390/cancers13020168)
Supplement: Supplementary file 1 [file cancers-13-00168-s001.pdf]

Supplementary Material: A Comprehensive Analysis of Baseline Clinical Characteristics and Biomarkers Associated with Outcome in Advanced Melanoma Patients Treated with Pembrolizumab

Gil Awada, Yanina Jansen, Julia Katharina Schwarze, Jens Tijtgat, Lennert Hellinckx, Odrade Gondry, Sim Vermeulen, Sarah Warren, Kelly Schats, Pieter-Jan van Dam, Mark Kockx, Marleen Keyaerts, Hendrik Everaert, Teofila Seremet, Anne Rogiers and Bart Neyns

Table S1. Scores in the NanoString PanCancer IO360 gene expression profiling panel.

TIS: tumor inflammation score
APM: antigen-presenting machinery
Apoptosis
ARG1: arginase 1
B7-H3: B7 homolog 3
CD45
CD8 T Cells
CTLA4: cytotoxic T-lymphocyte associated antigen-4
Cytotoxic Cells
Cytotoxicity
DC: dendritic cells
Endothelial Cells
Exhausted CD8
Glycolytic activity
Hypoxia
IDO1: indoleamine 2,3-dioxygenase 1
IFN Downstream: interferon downstream signaling
IFN Gamma: interferon gamma
IL10: interleukin-10
Immunoproteasome
Inflammatory Chemokines
Lymphoid
Macrophages
MAGEs: melanoma-associated antigens
Mast Cells
MHC2: major histocompatibility complex 2
Myeloid
Myeloid Inflammation
Neutrophils
NK CD56dim
NK Cells: natural killer cells
NOS2: Nitric oxide synthase 2
PD-1: programmed cell death 1
PD-L1: programmed cell death ligand 1
PD-L2: programmed cell death ligand 2
Proliferation
Stroma
T Cells
TGF-Beta: transforming growth factor beta
TH1 Cells: T helper 1 cells
TIGIT: T cell immunoreceptor with Ig and ITIM domains
Treg: regulatory T cells
MMR Loss: mismatch repair loss
Hypermutation
MSI Predictor: microsatellite instability predictor
APM Loss: antigen-presenting machinery loss
JAKSTAT Loss

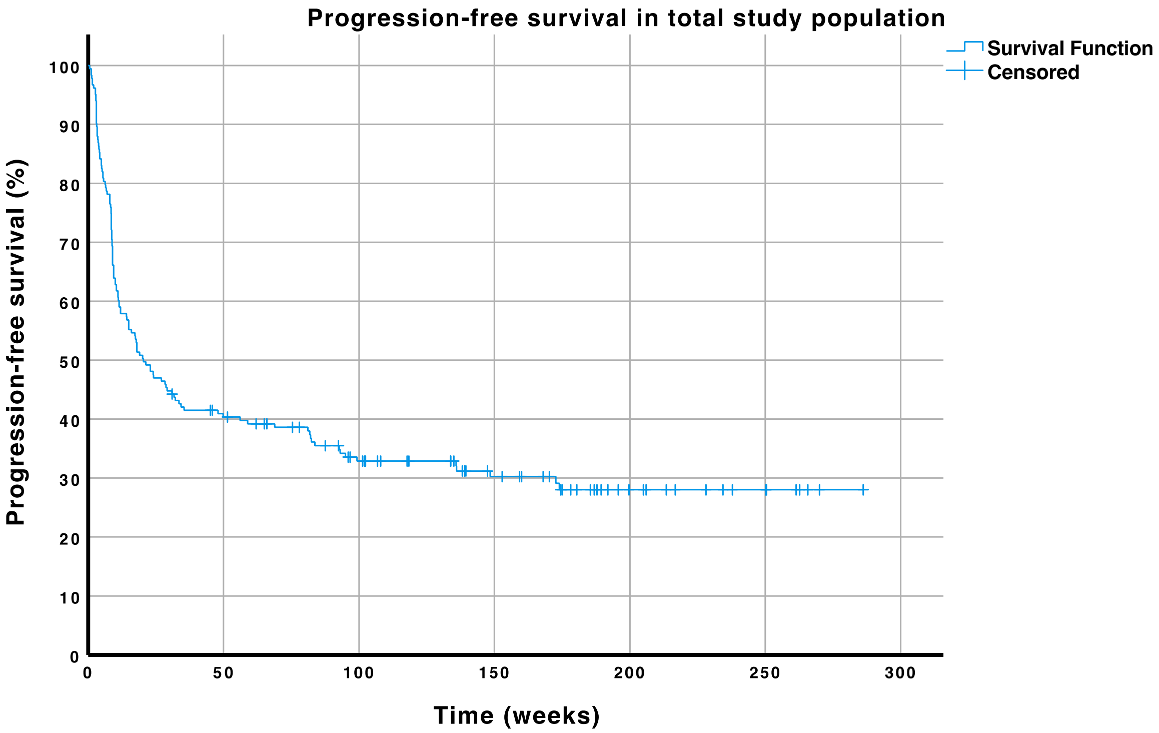

Figure S1. Progression-free survival in the total study population (n = 183).

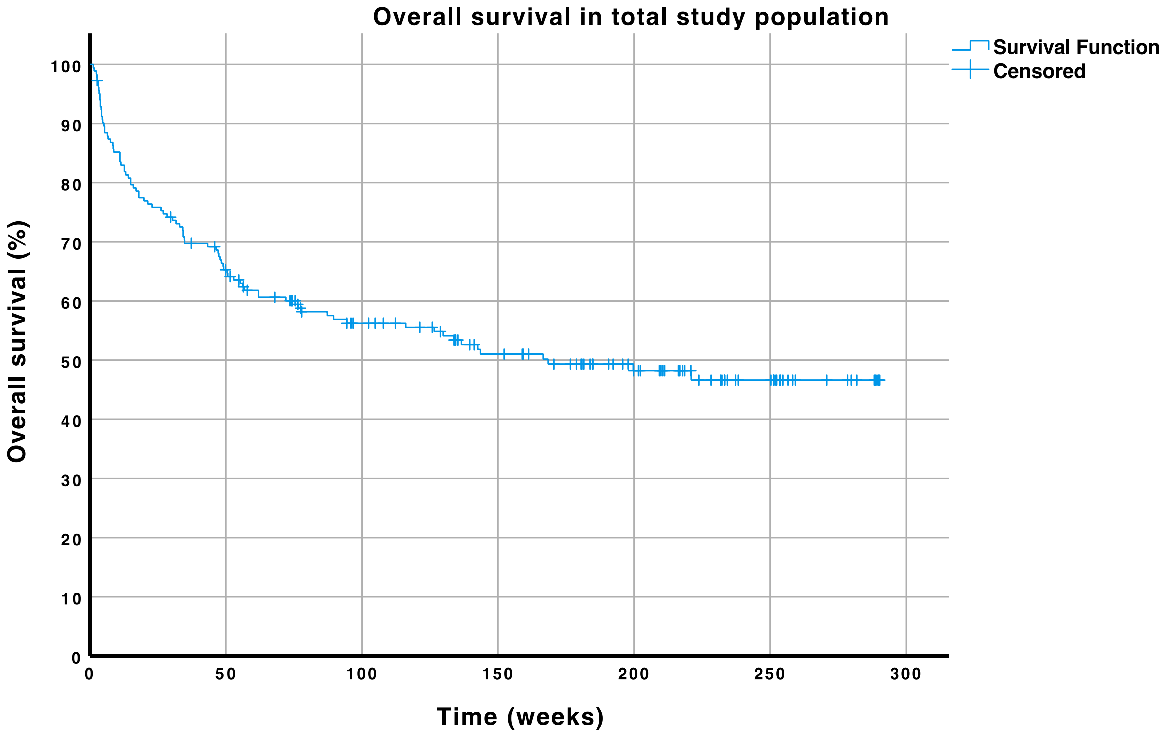

Figure S2. Overall survival in the total study population (n = 183).

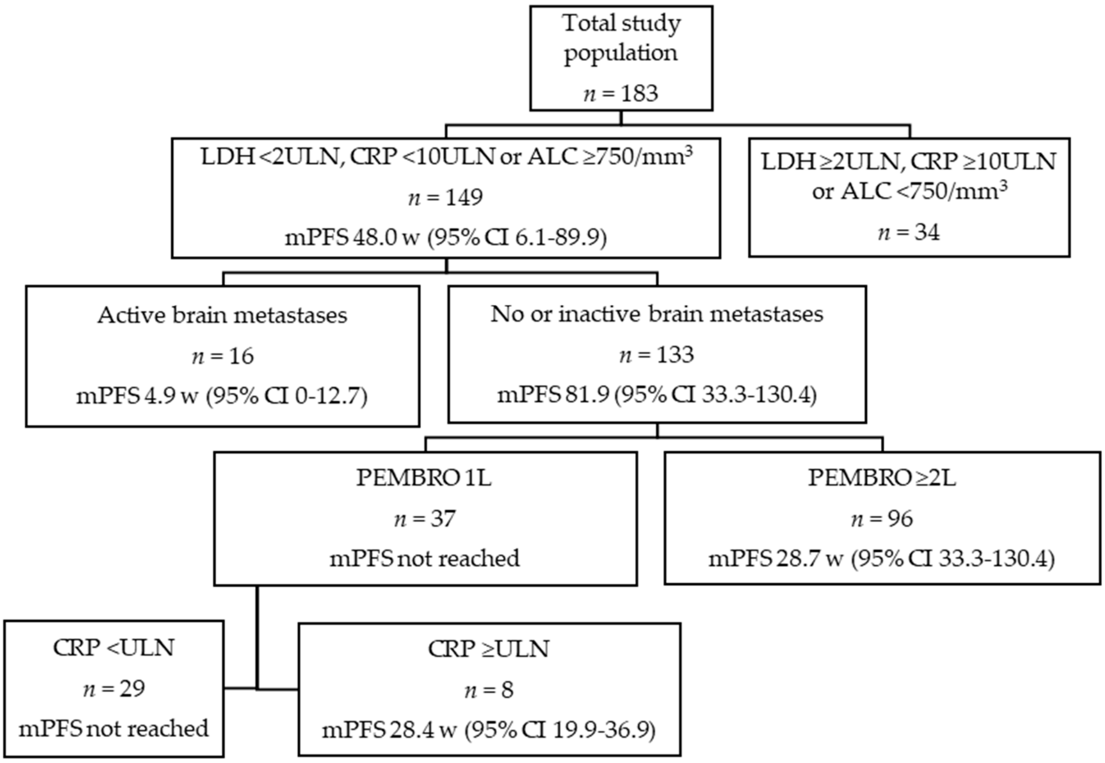

Figure S3. Recursive partitioning analysis in the population of patients with CRP <10ULN, LDH <2ULN or ALC ≥750/mm^3 (n = 149) with regards to progression-free survival. Abbreviations: 95% CI: 95% confidence interval; ALC: absolute lymphocyte count; CRP: C-reactive protein; LDH: lactate dehydrogenase; mPFS: median progression-free survival; PEMBRO 1L: pembrolizumab as first-line treatment; PEMBRO ≥2L: pembrolizumab as second line or later treatment; ULN: upper limit of normal; w: weeks.

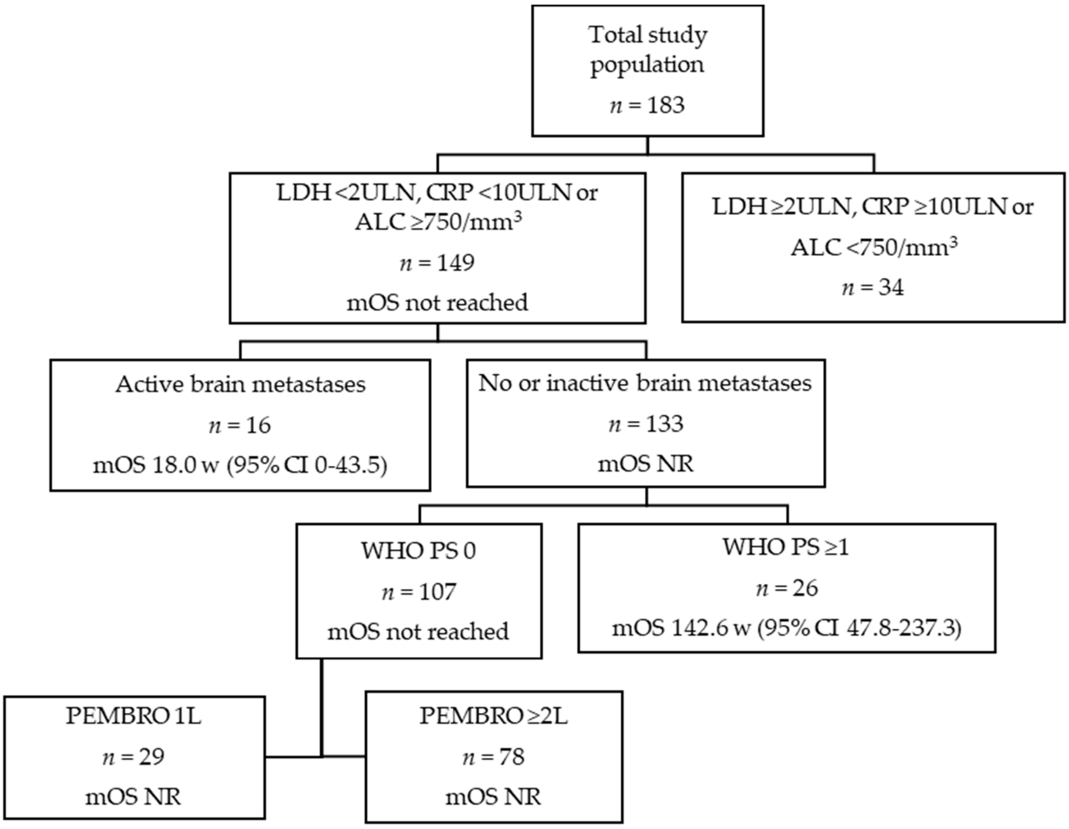

Figure S4. Recursive partitioning analysis in the population of patients with CRP <10ULN, LDH <2ULN or ALC ≥750/mm^3 (n = 149) with regards to overall survival. Abbreviations: 95% CI: 95% confidence interval; ALC: absolute lymphocyte count; CRP: C-reactive protein; LDH: lactate dehydrogenase; mOS: median overall survival; PEMBRO 1L: pembrolizumab as first-line treatment; PEMBRO ≥2L: pembrolizumab as second line or later treatment; ULN: upper limit of normal; w: weeks; WHO PS: World Health Organization Performance Status.

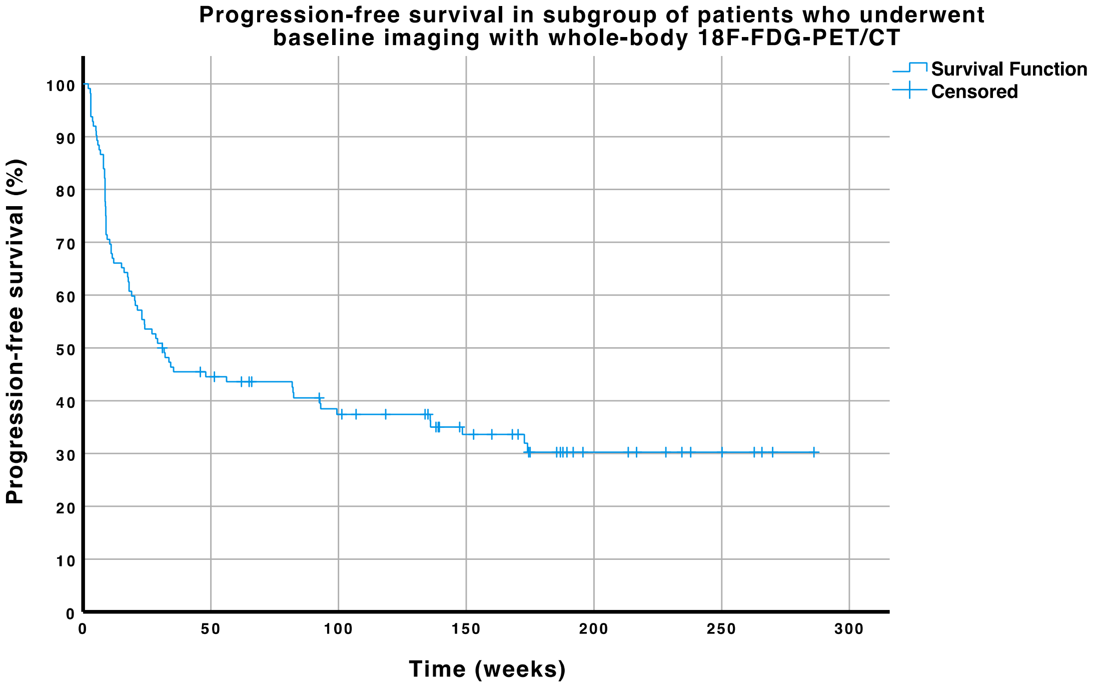

Figure S5. Progression-free survival in the subgroup of patients who underwent baseline imaging with whole-body ^{18}F-FDG-PET/CT ($n = 112$). Abbreviations: ^{18}F-FDG-PET/CT: 18-fluorodeoxyglucose positron emission tomography/computed tomography.

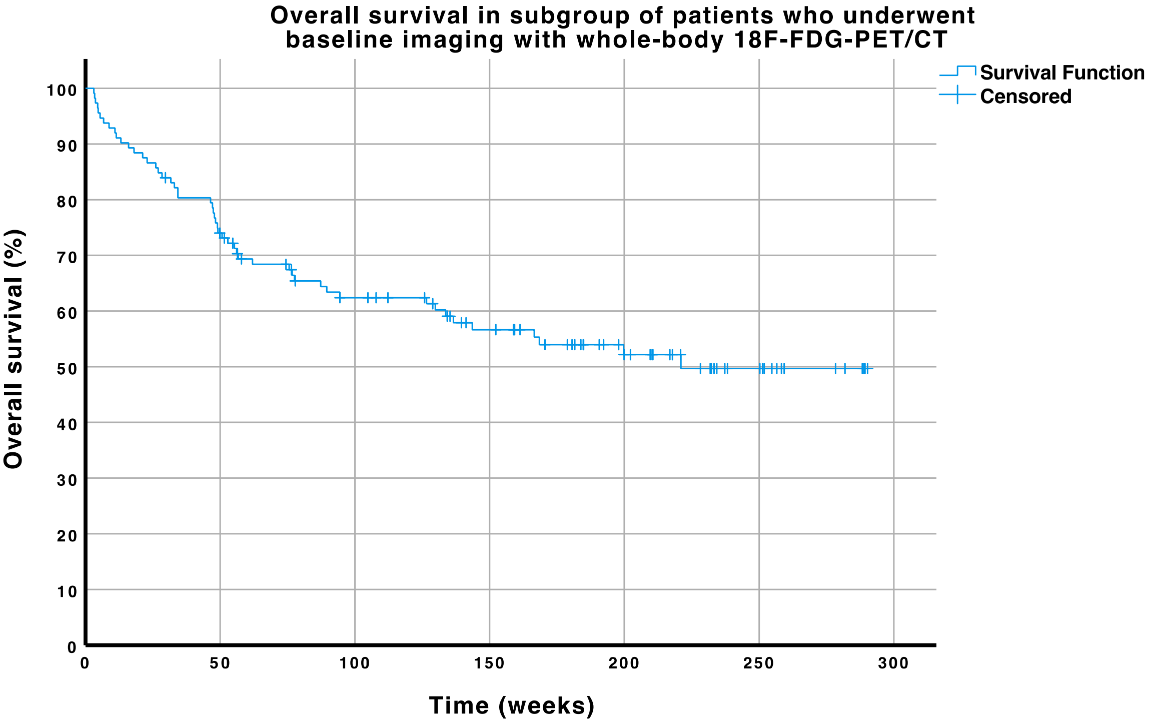

Figure S6. Overall survival in the subgroup of patients who underwent baseline imaging with whole-body ^{18}F-FDG-PET/CT ($n = 112$). Abbreviations: ^{18}F-FDG-PET/CT: 18-fluorodeoxyglucose positron emission tomography/computed tomography.

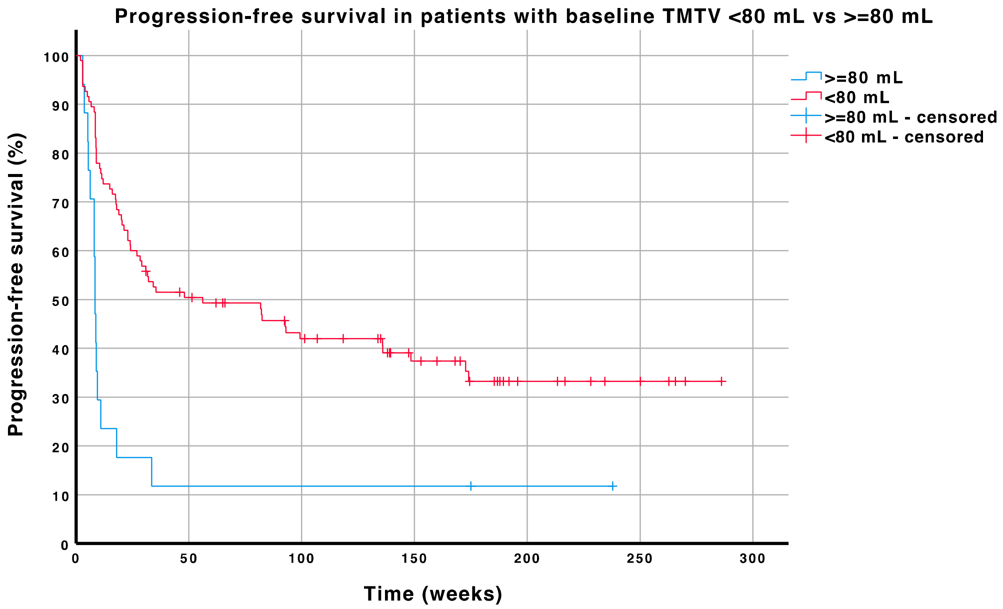

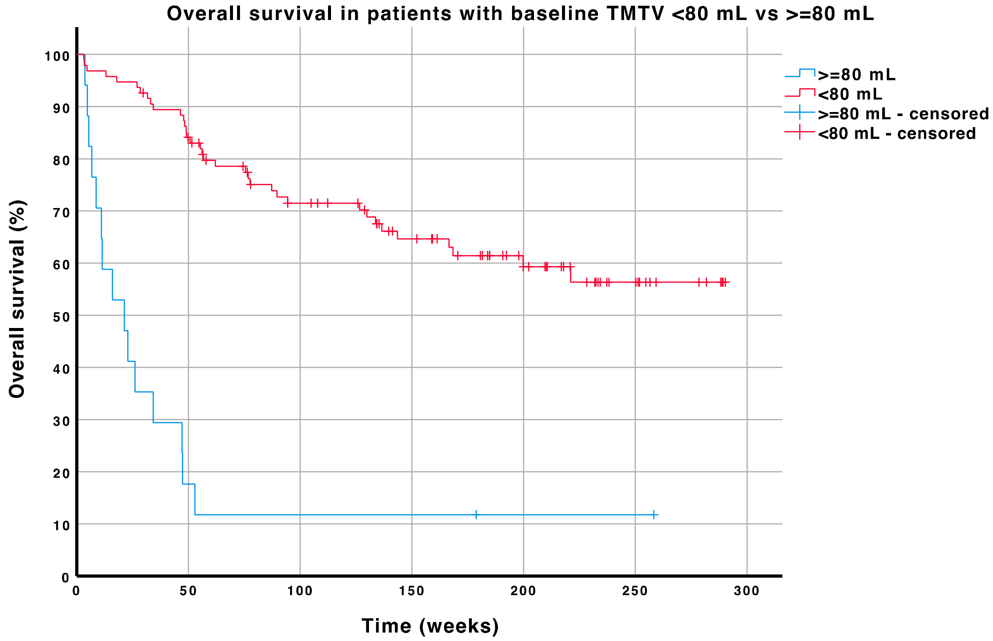

Figure S7. Progression-free and overall survival in the subgroup of patients who underwent baseline imaging with whole-body ^{18}F-FDG-PET/CT with baseline TMTV of <80 mL (*n* = 95) versus ≥80 mL (*n* = 17). Abbreviations: ^{18}F-FDG-PET/CT: 18-fluorodeoxyglucose positron emission tomography/computed tomography; TMTV: total metabolic tumor volume.

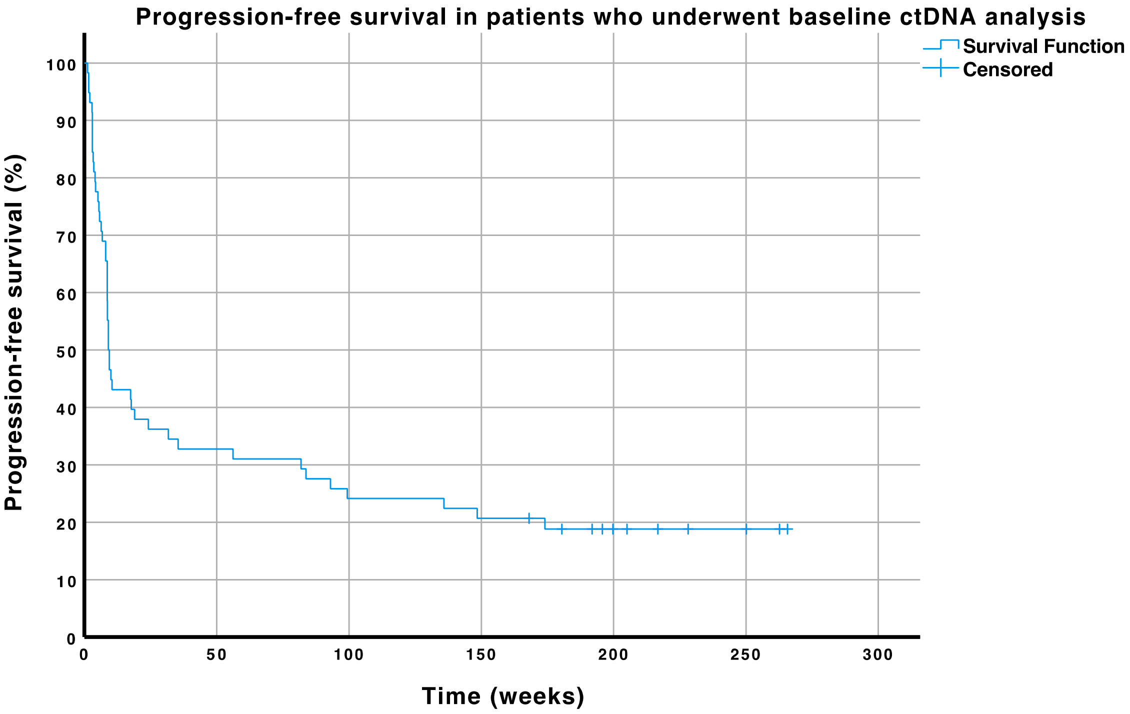

Figure S8. Progression-free survival curve in the subgroup of patients who underwent baseline ctDNA analysis (n = 58). Abbreviations: ctDNA: circulating tumor DNA.

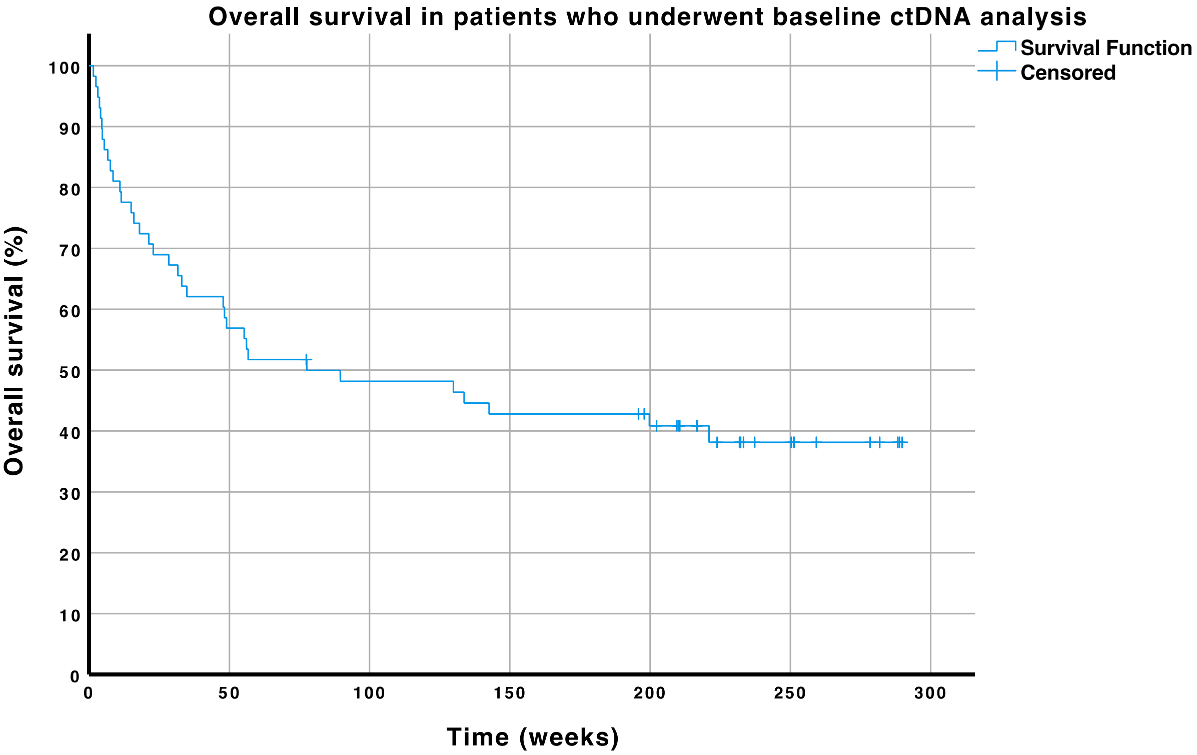

Figure S9. Overall survival curve in the subgroup of patients who underwent baseline ctDNA analysis (n = 58). Abbreviations: ctDNA: circulating tumor DNA.

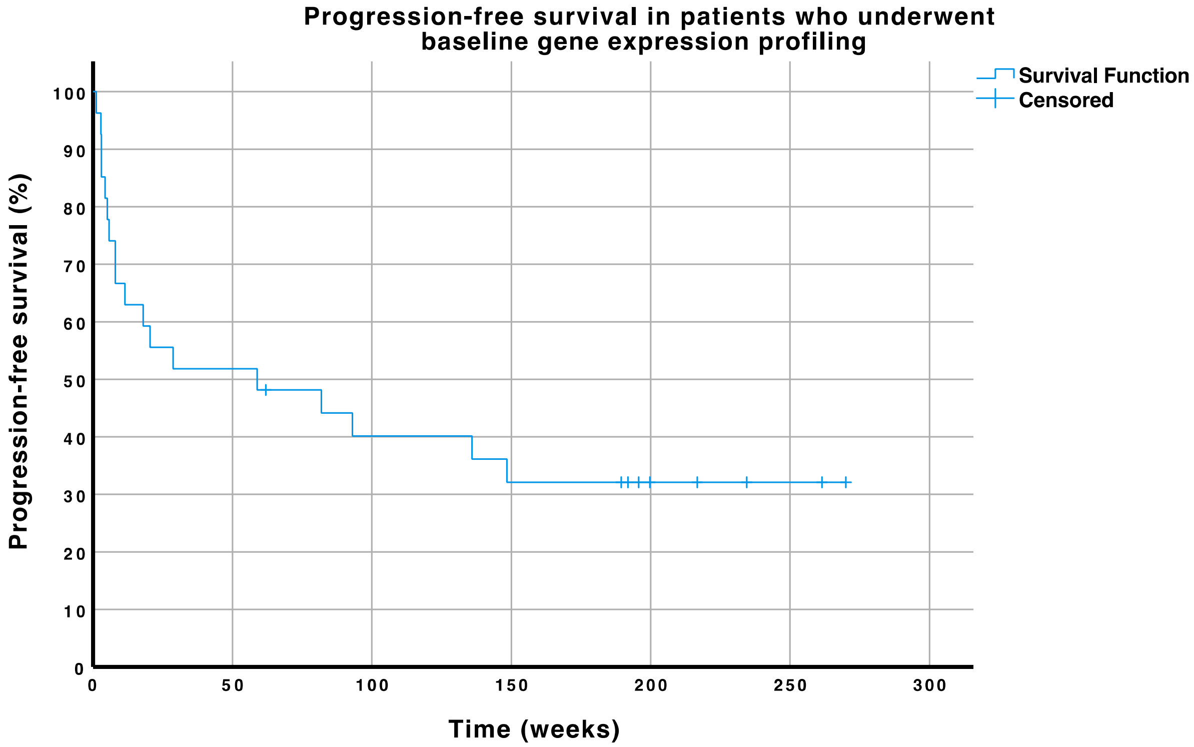

Figure S10. Progression-free survival curve in the subgroup of patients who underwent baseline gene expression profiling on tumor tissue ($n = 27$).

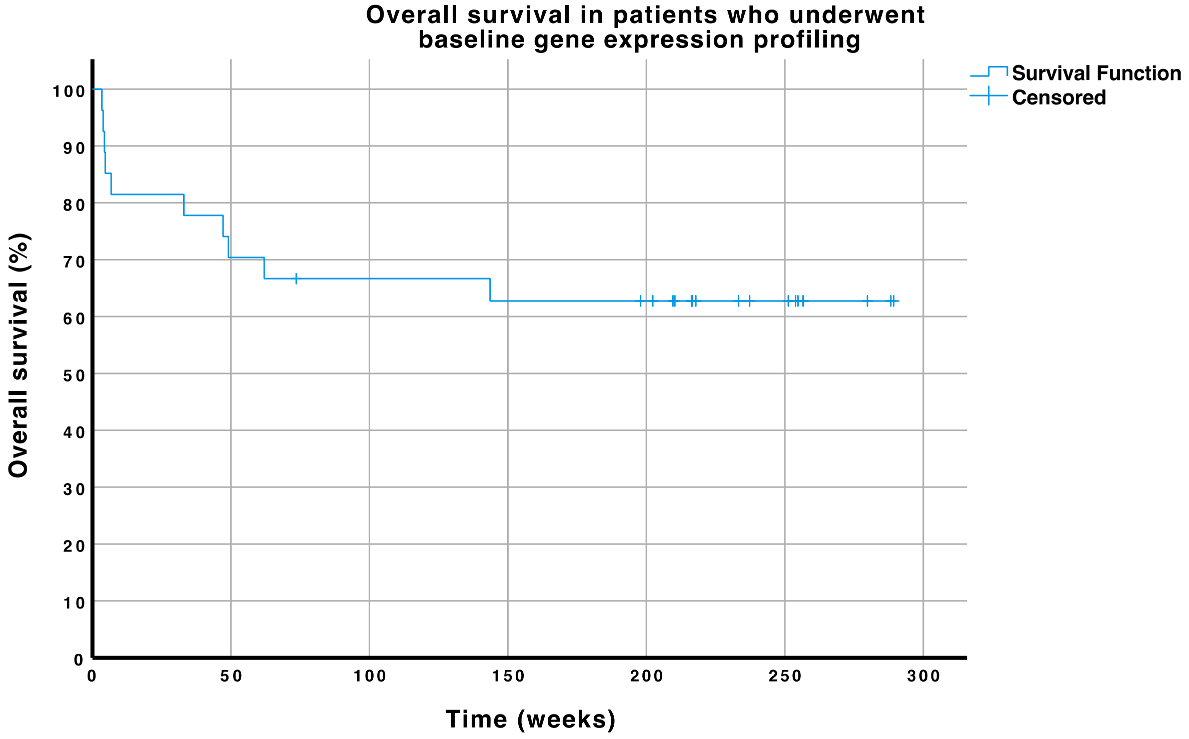

Figure S11. Overall survival curve in the subgroup of patients who underwent baseline gene expression profiling on tumor tissue ($n = 27$).
